# Baseplate Component TssK and Spatio-Temporal Assembly of T6SS in *Pseudomonas aeruginosa*

**DOI:** 10.3389/fmicb.2019.01615

**Published:** 2019-07-18

**Authors:** David Liebl, Mylène Robert-Genthon, Viviana Job, Valentina Cogoni, Ina Attrée

**Affiliations:** ^1^Univ. Grenoble Alpes, CNRS, Bacterial Pathogenesis and Cellular Responses, ERL 5261, INSERM, UMR-S 1036, CEA, Grenoble, France; ^2^Univ. Grenoble Alpes, CEA, CNRS, Institut de Biologie Structurale (IBS), Grenoble, France

**Keywords:** molecular nanomachine, T4 bacteriophage, bacterial competition, T6SS, baseplate assembly

## Abstract

The Gram-negative bacteria use the contractile multi-molecular structure, called the Type VI Secretion System (T6SS) to inject toxic products into eukaryotic and prokaryotic cells. In this study, we use fluorescent protein fusions and time-lapse microscopy imaging to study the assembly dynamics of the baseplate protein TssK in *Pseudomonas aeruginosa* T6SS. TssK formed transient higher-order structures that correlated with dynamics of sheath component TssB. Assembly of peri-membrane TssK structures occurred *de novo* upon contact with competing bacteria. We show that this assembly required presence of TagQ-TagR envelope sensors, activity of PpkA kinase and anchoring to the inner membrane *via* TssM. Disassembly and repositioning of TssK component was dependent on PppA phosphatase and indispensable for repositioning and deployment of the entire contractile apparatus toward a new target cell. We also show that TssE is necessary for correct elongation and stability of TssB-sheath, but not for TssK assembly. Therefore, in *P. aeruginosa*, assembly of the TssK-containing structure relays on the post-translational regulatory envelope module and acts as spatio-temporal marker for further recruitment and subsequent assembly of the contractile apparatus.

## Introduction

Gram-negative bacteria deploy a number of complex envelope-embedded multi-protein nanomachines adjusted exclusively for protein export across bacterial membrane and interaction with eukaryotic and prokaryotic species (Filloux et al., 2008; Hayes et al., 2010). One of these is Type VI secretion system (T6SS), originally identified in *Vibrio cholerae* using *Dictyostelium* host (Pukatzki et al., 2006). The T6SS is a versatile protein exporting machinery implicated in virulence as well as competition and cooperation in microbial communities and it is capable of targeting both bacteria and eukaryotic cells (Alcoforado Diniz et al., 2015; Bleves, 2016; Jani and Cotter, 2010). Thirteen essential core proteins are conserved in all T6SSs (Shalom et al., 2007; Cascales, 2008; Filloux et al., 2008; Boyer et al., 2009); the membrane associated complex TssJ-TssL-TssM, the baseplate proteins TssE, TssF, TssG, and TssK, the bacteriophage-related puncturing complex composed of the tube (Hcp), the tip/puncturing device VgrG, and the contractile sheath structure (TssB and TssC) (Coulthurst, 2013; Ho et al., 2014; Cianfanelli et al., 2016). Finally, the starfish-shaped dodecameric protein, TssA, limits contractile sheath polymerization at its distal part when TagA captures TssA (Planamente et al., 2016; Santin et al., 2018).

T6SS function involves a highly dynamic process of TssB/TssC sheath assembly, contraction and disassembly (Basler and Mekalanos, 2012; Basler et al., 2012; Forster et al., 2014), where recycling of the sheath subunits depends on ClpV ATPase (Bonemann et al., 2009; Pietrosiuk et al., 2011). Analogous to the T4 bacteriophage tail structure, the T6SS sheath assembles around an inner Hcp tube with membrane-puncturing device (VgrG) positioned at its proximal end (Leiman et al., 2009; Shneider et al., 2013; Silverman et al., 2013). Current model assumes that the positioning and contraction of the apparatus and subsequent ejection of effectors is strictly dependent on transient yet firm anchoring of the contractile device to precise loci within the bacteria membrane. Upon activation of the system, certain components such as Hcp and VgrG, could be exchanged between neighboring bacteria to launch rapid response to external stimuli (Vettiger and Basler, 2016).

Biogenesis of T6SS baseplate start with the association of two molecules of TssF, one molecule of TssG and two TssK trimers to form a stable complex, that then oligomerize further around the VrgG hub (Cherrak et al., 2018; Park et al., 2018). The composition of the whole 1.15 megadalton baseplate is proposed to be (TssE)_6_-(TssF)_12_-(TssG)_6_ with eighteen TssK subunits (Nazarov et al., 2018) connecting the whole assembly to the membrane-embedded 1.7 megadalton TssJLM complex (Brunet et al., 2015; Durand et al., 2015), where it stabilizes and initiates tube/sheath polymerization probably through interactions with TssE, as it is the case for its homolog gp25 in the phage (Yap et al., 2010; Nazarov et al., 2018).

Although the structural components of the T6SS machinery are conserved in several organisms, regulatory pathways controlling the expression and assembly of the apparatus differ. *Pseudomonas aeruginosa*, a major human opportunistic pathogen and causative agent of chronic infections in cystic fibrosis patients possesses three T6SS-encoding loci and a number of effector-encoding genes spread through the genome (Filloux et al., 2008). The machinery encoded by the Hcp secretion island I-encoded T6SS (H1-T6SS) (Mougous et al., 2006) is used for bacterial warfare and can translocate multiple effectors into target bacteria (Russell et al., 2011, 2012). The expression and activity of H1-T6SS is controlled at the transcriptional and translational level by a regulatory network including membrane sensors (RetS, LadS, and GacS) (Brencic and Lory, 2009; Brencic et al., 2009), small regulatory RNAs (Brencic and Lory, 2009; Brencic et al., 2009) and di-cGMP signaling (Moscoso et al., 2011). At the post-translational level, eukaryotic-like threonine kinase/phosphatase pair (PpkA-PppA) and a signal-transmission module (TagQ-TagR-TagS-TagT) embedded within the bacterial envelope are required for the T6SS activity (Mougous et al., 2007; Hsu et al., 2009; Basler et al., 2013). The Tag proteins are found in the so-called “defensive” T6SS and modulate the T6SS activity in response to an attack from a competitor, while the “offensive” system is always active, ready to fire on any surrounding bacteria as in the case of *V. cholera* (Basler et al., 2013; Ostrowski et al., 2018).

The aim of this study was to examine the role of signal-transmission module and threonine kinase/phosphatase pair on initial T6SS baseplate assembly. To that purpose, we used quantitative time-lapse microscopy imaging to investigate the spatio-temporal recruitment and assembly of two baseplate proteins, TssK, TssE and the sheath component, TssB. Although the fusion constructs compromised the very last step of T6SS action, i.e., prey killing, they reflected the dynamics of all initial steps of machinery function, including sensing, baseplate and contractile apparatus assembly and firing. As the exploring behavior of TssK/TssB fusions was possible only in the wild-type background, we propose that assembly of baseplate/sheet structure still required a pool of endogenous (non-tagged) TssK/TssB to co-assemble with exogenous (tagged) TssK/TssB. Our results show dynamic assembly of the T6SS baseplate structure that depends on *P. aeruginosa* post-translational signaling cascade, as well as the key functions the baseplate provides for assembly and relocation of the T6SS nanomachinery to a site of contact with competing bacteria.

## Results

### TssK Assembles Into Peri-Membrane Higher-Order Structures in Response to TagQRST-Mediated Activation of T6SS

**FIGURE 1:**
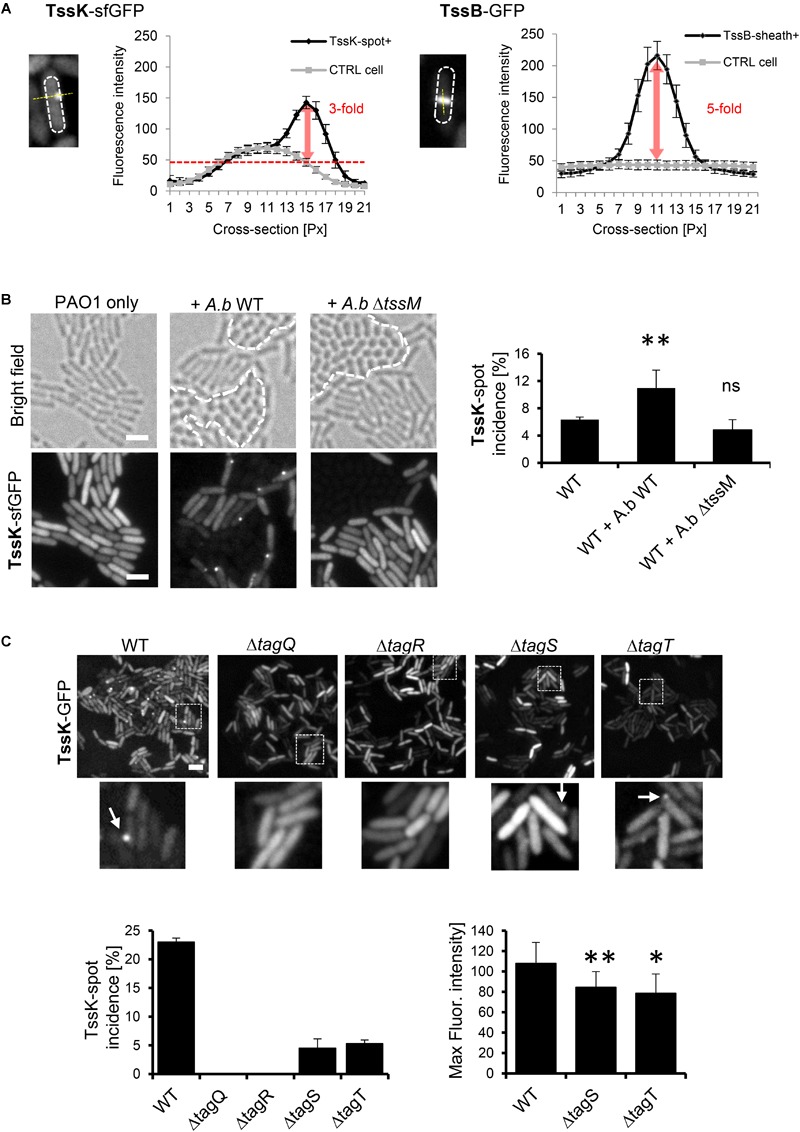
TssK assembles into discernible perimembrane structures in response to T6SS activation. **(A)** Fluorescent intensities of TssK-sfGFP rounded foci and TssB-GFP elongated rods observed in PAO1 strain after arabinose-induction. Note the 3-fold and 5-fold higher intensity compared to the cytoplasmic levels for TssK and TssB, respectively. **(B)** Assembly of TssK-sfGFP increases in mixed culture with wild type *A. baumannii* but not with T6SS-inactive *A. baumannii* mutant Δ*tssM*. Note that *A. baumannii* can be discriminated from *P. aeruginosa* by its rounded-cell morphology in bright field images (borders between two species are depicted by dashed lines). Representative bright field and fluorescent images are shown with quantification of TssK-spot incidence. Average and SD values were calculated for each group from 4 to 6 ROI each containing between 400 and 1000 of cells. Two-tail *t*-test, ^∗^*P* = 0.038. **(C)** Role of TagQRST sensor module in TssK assembly. Fluorescence images are shown for each *tag* mutant. Histograms showing quantification of TssK-spot (white arrows in images) incidence and fluorescence intensity measurements of TssK-sfGFP structures are shown below. No spots of TssK-sfGFP were found in Δ*tagQ* and Δ*tagR* mutants and deletion of *tagS* or *tagT* lead to a significant reduction of the incidence of TssK-spot formation. Note that fluorescence intensity of TssK-sfGFP spots in Δ*tagS* and Δ*tagT* mutants was significantly decreased. Average and SD values were calculated from 20 spots for each sample. Two-tail *t*-test, ^∗∗^*P* = 0.03 (Δ*tagS*) and ^∗^*P* = 0.09 (Δ*tagT*). ns, non significant. Bar = 2 μm.

To examine whether the environmental signals, transmitted by TagQRST, influence the baseplate assembly we engineered fusions between genes encoding several baseplate proteins (*tssE, tssF, tssG* and *tssK*) with *sfGFP* and *tssB-GFP*. The plasmids carrying the fusion under arabinose-inducible promoter were introduced in the wild-type *P. aeruginosa* strain PAO1. We were unable to exploit any florescent signal with TssG-sfGFP and TssF-sfGFP. However, in a subpopulation of bacteria, TssK-sfGFP assembled into discernible foci localized to the bacterial periphery, reminiscent to TssK foci observed in *Escherichia coli* (Brunet et al., 2015; Cherrak et al., 2018), whereas TssB-GFP assembled into elongated rod-like structures with substantially higher fluorescence intensity relative to TssK foci (Figure 1A). In *P. aeruginosa*, T6SS assemble preferentially upon the attack by neighboring bacteria in a behavior named “T6SS dueling” (Basler and Mekalanos, 2012; Basler et al., 2013). We analyzed *P. aeruginosa* cells expressing TssK-sfGFP grown in presence of *Acetinobacter baumannii* and found that spots formed by TssK-sfGFP were mostly positioned specifically toward contacts with *A. baumannii* (Figure 1B), as would be expected for a “defensive” T6SS apparatus. Notably, quantification revealed that incidence of TssK-sfGFP spots in *P. aeruginosa* increased significantly in mixed culture with wild type *A baumannii* but not with a *tssM* mutant (*Ab*Δ*tssM*) with an inactive T6SS (Weber et al., 2013); showing that TssK-sfGFP spot formation correlates specifically with a response of *P. aeruginosa* to T6SS-mediated attack by competitor bacteria. Thus, TssK has the capacity to assemble from the cytosolic pool into perimembrane higher order structures with the incidence that increases specifically during interaction with competitor bacteria. In competition assays with *E. coli* DH5α, the killing capacity of the PAO1 strain expressing TssK-sfGFP toward the pray was diminished compared to PAO1 (wild type) (Supplementary Figure S1). This suggest that TssK-sfGFP incorporates within the baseplate with native TssK but limits the capacity of T6SS to inject effectors. Similar dominant-negative effect on T6SS activity was already observed for single domains of TssK in enteroaggregative *E. coli* (Cherrak et al., 2018).

However, the TssK-sfGFP protein fusion construct could be used to study T6SS assembly kinetics, as demonstrated in the following experiments.

A unique feature of post-translational regulation of T6SS activity in *P. aeruginosa* is the sensory module composed of TagQ, TagR, TagS, and TagT proteins encoded together with other T6SS core components within the HSI-1 locus (Cascales, 2008; Filloux et al., 2008; Boyer et al., 2009). TagQ is an outer-membrane lipoprotein required for recruitment of periplasmic TagR which in turn activates a Threonine Protein Phosphorylation (TPP) pathway promoting assembly of the T6SS apparatus (Mougous et al., 2007; Hsu et al., 2009). TagS and TagT form a classical ABC transporter embedded within the inner membrane of bacteria, and all four proteins are necessary for optimal T6SS activation *in vitro* (Casabona et al., 2013). We used previously characterized Δ*tagQ*, Δ*tagR*, Δ*tagS* and Δ*tagT* mutants (Casabona et al., 2013) and analyzed TssK-sfGFP localization and its incidence in these cells mixed with *A. baumannii*. Unlike the wild type, no TssK-assemblies were detected in Δ*tagQ* and Δ*tagR* mutants (Figure 1C). Interestingly, deletion of *tagS* or *tagT* did not lead to complete block of TssK assembly, yet significantly reduced the incidence of TssK-assembling cells so as the overall spot size (fluorescence intensity) of TssK-structures relative to the control (Figure 1C). Consequently, we found that assembly of TssK into a baseplate structure within a particular site at the plasma membrane is dependent on TagQ and TagR. We established that presence of TagS and TagT was dispensable for TssK assembly but may have modulatory/accessory function in the process of TssK recruitment into functional entities, in agreement with previous findings that TagT was required for “dueling” behavior and reposition of the apparatus toward neighboring sister cells (Basler et al., 2013). We conclude that observed TssK-sfGFP foci represent protein complexes within T6SS apparatus that are assembled in post-translational manner and can be used as a marker for monitoring T6SS baseplate assembly.

### TssK Undergoes Dynamic Assembly-Disassembly

**FIGURE 2:**
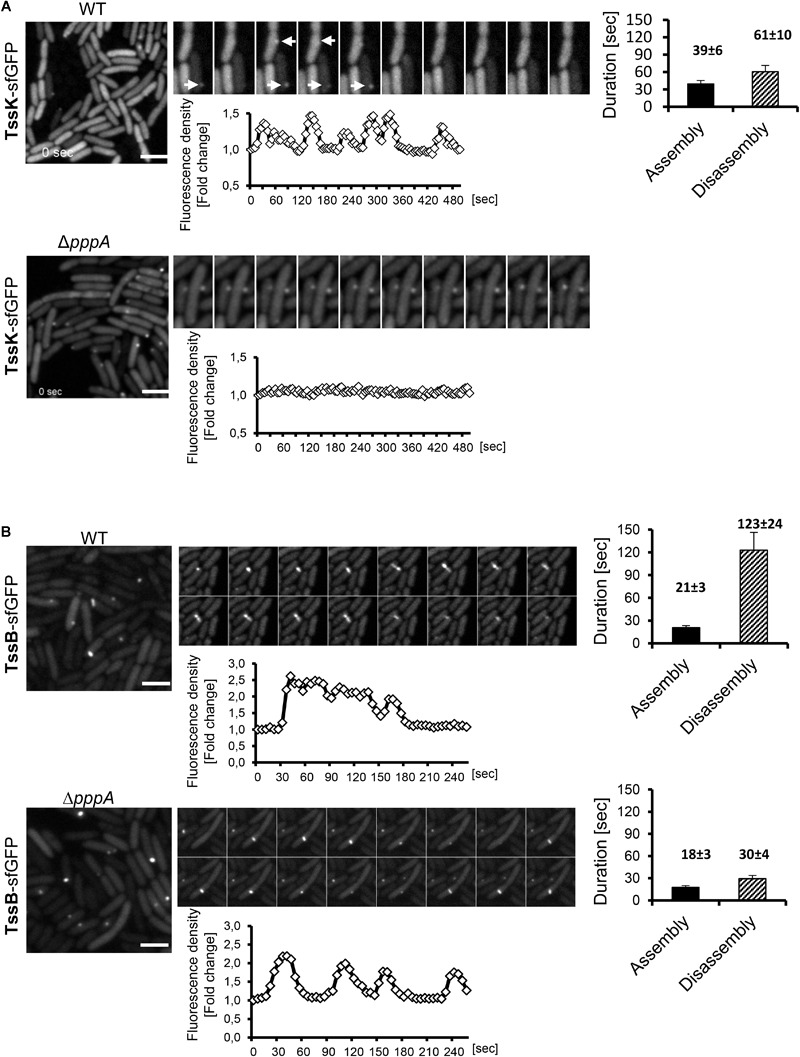
Assembly of TssK baseplate component requires activity of PpkA kinase while disassembly and repositioning is dependent on PppA phosphatase. Time-lapse series of *P. aeruginosa* PA01 (WT) and PppA-deficient mutant (Δ*pppA*) expressing TssK-sfGFP **(A)** or TssB-GFP **(B)**. Series of fluorescence images are shown (10 s between frames) with kinetic measurements of relative fluorescence intensity in ROI containing TssK-sfGFP/TssB-GFP structures (below) and quantification of an average duration of assembly/disassembly of these structures (on right). Bars = 2 μm. **(A)** In wild type cells, individual TssK-sfGFP structures (white arrows) assemble/disassemble within an average 100 ± 10 s. Note that lack of PppA impaired recycling and re-positioning of TssK structures but not their assembly *per se*. **(B)** A single round of assembly-contraction-disassembly of individual TssB-GFP sheaths in wild type occurs within an average 144 ± 24 s period. Note that in Δ*pppA*, the TssB-sheath assembles repeatedly at the same location and exhibits an aberrant kinetics with significantly faster rate of disassembly (30 ± 4 s) relative to the wild type control (123 ± 24 s).

We then analyzed *P. aeruginosa* cells expressing TssK-sfGFP by time-lapse imaging to examine its dynamic features including assembly/disassembly and/or lateral displacement or motility. Real-time detection of transient appearance-growth-decay-disappearance of discernible perimembrane-localized fluorescent spot(s) was measured as described in Section “Materials and Methods,” and reported as assembly/disassembly. Intensity measurements started prior structure appearance (except for TssK in *pppA* mutant) where values at T0 (normalized to 1) correspond to fluorescence intensity of cytosolic pool of the fusion protein. We typically observed multiple rounds of assembly disassembly occurring at the same location before assembly was detected at another location of the same bacteria. Interestingly, no more than one TssK-sfGFP spot was found per single cell at a given time. Kinetic measurements of fluorescence intensity revealed that assembly of the TssK-sfGFP occurred within 39 ± 6 s whereas disassembly of the structures lasted in average 61 ± 10 s (Figure 2A), implying that these transient structures undergo frequent assembly-disassembly as a dynamic component of T6SS baseplate structure.

Downstream of TagQRST sensory module, the T6SS activity is post-translationally regulated by the threonine kinase PpkA whose activity is antagonized by the phosphatase PppA (Mougous et al., 2007; Ostrowski et al., 2018). We detected TssK-sfGFP assemblies in the Δ*pppA* mutant but never in the Δ*ppkA* mutant of *P. aeruginosa* (Figure 2A and Supplementary Figure S2), despite the same expression levels of corresponding constructs as revealed by immunoblot analysis (Supplementary Figure S3). Similar result was reported for TssB and Fha foci in Δ*pppA* and Δ*ppkA* mutants in *S. marcescens* (Ostrowski et al., 2018). Kinetic measurements showed that in Δ*pppA*, structures formed by TssK-sfGFP were static, permanently retained at one perimembrane site without *de novo* assembly at another location (Figure 2A). This suggests that the dephosphorylation of Fha, a target of PpkA kinase and PppA dephosphatase, is necessary for repositioning of the TssK-containing baseplate, but not for its assembly *per se*. Otherwise, PppA could have another, yet unidentified target. To note, in the wild-type *E. coli*, TssK foci also seem to be static at a given time but several of them were detected per cell, which may be explained by the absence of post-translational regulatory module in this system (Brunet et al., 2015).

To investigate how TssK dynamics relates to the assembly, contraction and disassembly of the contractile sheath in *P. aeruginosa* we first analyzed the sheath dynamics in *P. aeruginosa* expressing TssB-GFP construct. Similar to TssK, the TssB-GFP exhibited homogenous cytosolic localization in a vast majority of cells but elongated sheath-like structures were readily detected in mixed cultures with *A. baumanni*. As for TssK foci, we have never detected more than one TssB-sheath structure per individual cell at a given time. Quantitative measurements of assembly-disassembly revealed distinct kinetics features for TssB-sheaths, relative to TssK structures, where assembly occurred within 21 ± 3 s followed by disassembly at a slower rate of 123 ± 24 s resulting in an average 140 s lifetime for an individual TssB-structure (Figure 2B), similar to value found by others (Basler and Mekalanos, 2012; Basler et al., 2012). Kinetic profiles of TssK-baseplate in homogenous culture of *P. aeruginosa* were similar to corresponding profiles in competition conditions where the assembly of TssK structures was targeted preferentially toward the contact with competing *A. baumannii* (Supplementary Figure S4). Like TssK assemblies, several rounds of assembly-contraction of TssB-sheaths were detected at the same site of the bacteria prior complete disassembly of the structure. The dynamic feature of the contractile sheath (TssB-GFP) was not lost in *P. aeruginosa* mutant Δ*pppA* but led to significantly faster (likely premature) disassembly of the sheath relative to wild-type (30 ± 4 vs. 123 ± 24 s, respectively).

We conclude that similarly to contractile sheath component TssB, the baseplate component TssK also undergoes regulated periodic assembly and disassembly during deployment of the T6SS apparatus. This implies that in cells primed for T6SS activity, TssK does not assemble into multiple rigid baseplate structures per cell, but instead assembles transiently *ad hoc* at a specific site of the inner membrane toward the contact with neighbor attacking bacteria. We provide evidence that PppA phosphatase functions downstream of this TssK assembly and is essential for its disassembly and relocation to a new site for subsequent *de novo* assembly of the contractile apparatus.

### TssK Functions as a Spatio-Temporal Marker for Assembly of Contractile Apparatus

**FIGURE 3:**
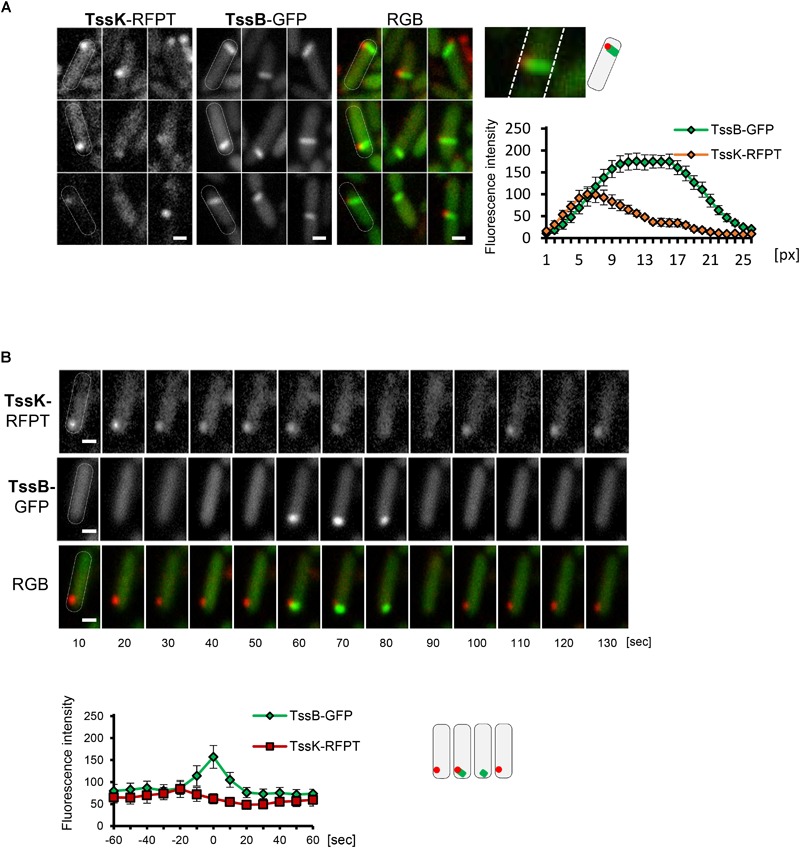
TssK functions as essential positional marker for recruitment, nucleation and assembly of contractile apparatus. **(A)** TssK assembles into discrete structure at the proximal tip of TssB-sheath. Several representative fluorescence images of WT cells co-expressing TssK-RFPT and TssB-GFP. Note that fully elongated TssB-sheath spans the entire width of bacteria. Fluorescence intensity profile of structures positive for both TssK-RFPT and TssB-GFP measured on line cross-section from proximal to distal pole with average values and SD calculated from 20 structures (*N* = 20). Schematic representation of bacteria with TssK (in red) and TssB (in green). **(B)** TssK and TssB assemblies within individual bacteria exhibit spatial correlation, but differential temporal co-localization. Time-lapse of fluorescence images of representative bacteria co-expressing TssB-GFP and TssK-RFPT (top panel). Note that TssK structure transiently disappears after the onset of TssB assembly at that site. Kinetics of fluorescence intensity measured at ROI containing nascent TssK/TssB structure indicates an interval (20 ± 10 s) between fluorescence maxima of TssK-RFPT and TssB-GFP. SD were calculated from ROI of 20 bacteria (*N* = 20). Bars = 0.5 μm. Schematic representation of bacteria from time lapse in B, with TssK (in red) and TssB (in green).

Because both, TssK-sfGFP structures and TssB-GFP sheaths exhibited similar yet distinct dynamic phenotype, we examined TssK as a spatio-temporal cue for recruitment of contractile sheath components TssB. We analyzed colocalization between TssK and TssB and measured the kinetics of their assembly-disassembly in *P. aeruginosa* co-expressing TssK-RFPT and TssB-GFP. Correspondent with our previous observations with individually expressed proteins, TssK only assembled into rounded spots at the periphery of bacteria, whereas TssB assembled into rod-like structure often spanning the entire width of the cell (in an extended conformation) (Figure 3A). It is noteworthy that in bacteria with discernible TssK and TssB assemblies the TssK structure co-localized exclusively with the proximal tip of the TssB structure at the perimembrane site (Figure 3A,B), in agreement with proposed baseplate localization and function as a platform for sheath polymerization (Brunet et al., 2015; Cianfanelli et al., 2016; Cherrak et al., 2018; Nazarov et al., 2018).

Importantly, in the wild-type strain co-expressing both fusions the incidence of discernible TssK structures at random time points (as captured on still images) was more frequent than that of TssB-sheaths and consequently a vast majority (95%) of TssB sheaths was marked by discernible TssK-structure, while only a subpopulation of cells with TssK structure (40%) also exhibited assembled TssB-sheath (not shown). This suggests that both components assemble sequentially rather than simultaneously into the apparatus and that only a fraction of TssK assemblies gives rise to TssB-sheath assembly, similarly to a study in *E. coli* (Santin et al., 2018). We then examined relative timing of TssK and TssB assembly by time-lapse analysis of individual bacteria followed by quantitative measurement of fluorescence intensity within a region of interest (ROI) covering the site of TssK/TssB assembly. Although the incidence of TssK and TssB assemblies exhibited a spatial correlation within individual cells, their temporal co-localization was mutually exclusive. Quantitative fluorescence intensity measurements also revealed consistent interval of 20 ± 10 s between kinetic fluorescence maxima of TssK and TssB assemblies (Figure 3B), suggesting that TssK component of the baseplate is involved in the initial stage of TssB-sheath nucleation rather than in subsequent steps of sheath elongation, contraction or disassembly.

### TssK Requires TssM to Trigger TssB-Assembly

**FIGURE 4:**
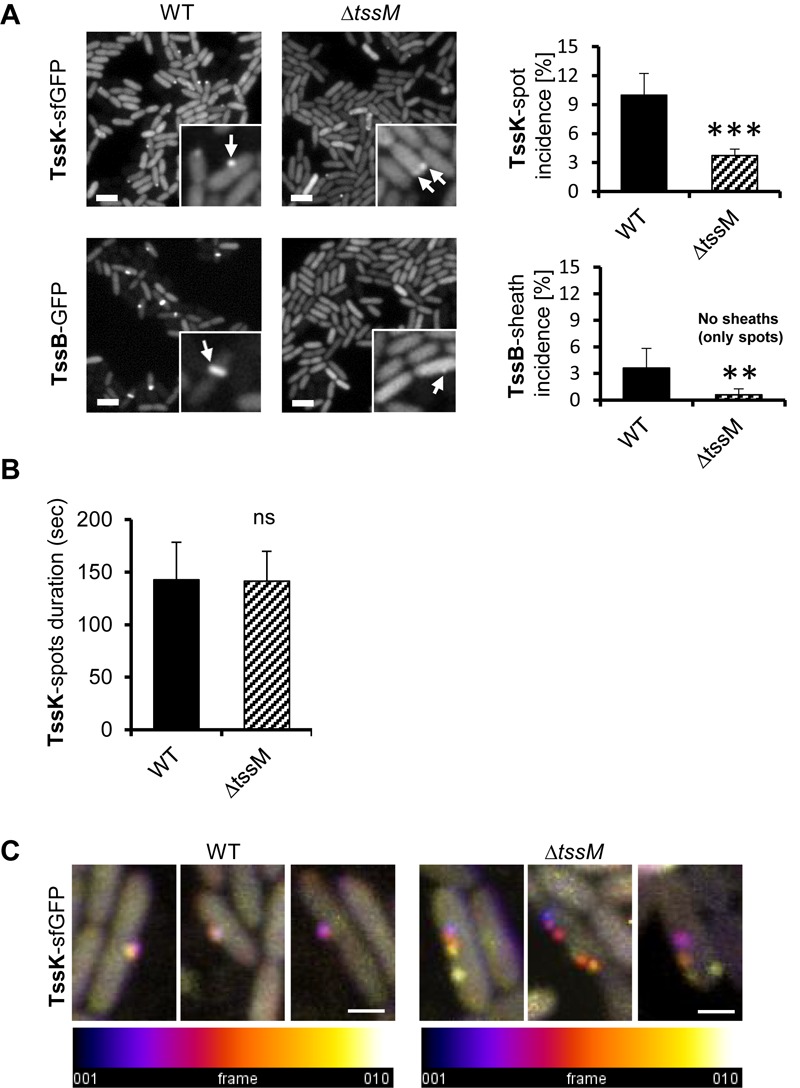
Assembly of TssK baseplate is essential but not sufficient to trigger TssB-assembly and requires stable anchor *via* TssM. **(A)** Assembly of TssK-baseplate and TssB-sheath in *P. aeruginosa* PAO1 lacking transmembrane component of T6SS apparatus, TssM. Representative fluorescence images (left) and quantification of TssK or TssB assemblies (right). Note that incidence of TssK structures (white arrows) in Δ*tssM* cells is significantly reduced, but not completely abolished, while elongated TssB-sheaths (white arrows) are only visible in PAO1 and only minor subpopulation of Δ*tssM* bacteria (<0.6%) was found with a spot-like structure of TssB-GFP. Two-tail *t*-test, ^∗∗∗^*P* = 0.002 (TssK) and ^∗∗^*P* = 0.015 (TssB). Bars = 2 μm. **(B)** Quantification of TssK-spot duration as a time between appearance and disappearance of discernible TssK-sfGFP structure over the background fluorescence intensity of the fusion construct. Note that kinetics of TssK-sfGFP baseplate assembly-disassembly is not impaired in Δ*tssM* relative to the WT cells (140 ± 30 s). Average values and SD were calculated from 30 cells (*N* = 30). Two-tail *t*-test. ns, non-significant. **(C)** Three representative time-lapse series (10 s between frames, 10 frames) of WT and Δ*tssM* cells expressing TssK-sfGFP. Each series were superimposed and color-coded (fire LUT) to visualize TssK structure stability/displacement over time. Note that TssK-structure in Δ*tssM* undergoes lateral displacement. Bars = 0.5 μm.

To determine the impact of the membrane component TssM on the kinetics of TssK assembly, we analyzed the incidence of TssK and TssB assemblies in *P. aeruginosa* PAO1Δ*tssM.* We found that although deletion of *tssM* significantly decreased the incidence of bacteria positive for TssK foci (from 10% in the parental strain to 3.7% in Δ*tssM*) the assembly of TssK structures and their localization to the membrane was not completely abolished (Figure 4A). In contrast, elongated TssB rod-shaped assemblies were detected only in wild-type bacteria and never in Δ*tssM.* We noted however, that in less than 0.6% of Δ*tssM* bacteria the TssB assembled into low-intensity round-shaped fluorescent spot(s) localizing to the membrane but these structures never extended into a shape of an extended sheath-like structure (Figure 4A). To assess possible impact of *tssM* deletion on dynamic properties of TssK we quantified an average time span of TssK structure (period between onset of assembly and complete disassembly/disappearance) by measuring the TssK-GFP fluorescence intensity in time-lapse of Δ*tssM* relative to wild-type bacteria. We found kinetics of TssK assembly-disassembly to be almost identical in both genetic backgrounds (140 ± 30 s per single round of assembly-disassembly) (Figure 4B). However, in a contrast to the wild-type, TssK-assemblies in Δ*tssM* often “detached” from the original perimembrane site and exhibited lateral movement along the membrane before their complete disassembly (Figure 4C).

Together, these results show that (i) TssM is not necessary for assembly of TssK *per se*, (ii) TssM is essential for stabilization of assembled TssK structure at a fixed perimembrane site and (iii) in the absence of TssM, the TssB protein is still recruited to the structure containing TssK, but the assembly of TssB-sheath is aborted (likely at the nucleation stage). This implies that TssM provides a necessary anchor, probably through the outer membrane lipoprotein TssJ (Felisberto-Rodrigues et al., 2011) for transient assembly of TssK-oligomeric structure. This could restrict its lateral movement along cell periphery, which in turn is required for subsequent recruitment, assembly and elongation of TssB-sheath.

### TssE Functions Downstream of TssK and Prevents Detachment of TssB-Sheath and Its Premature Disassembly

**FIGURE 5:**
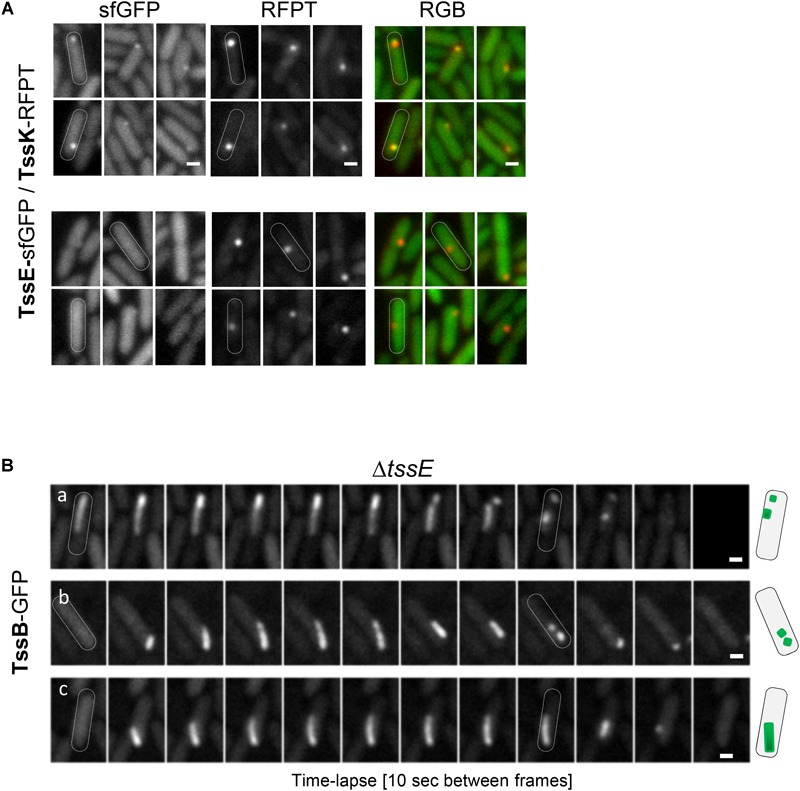
Role of TssE in baseplate assembly and elongation of TssB-sheath. **(A)** TssE-sfGFP assembles into discernible perimembrane structures and colocalizes with TssK in *P. aeruginosa* PAO1 co-expressing *tssE*-*sfGFP* with *tssK*-*RFPT* (upper panel). In some cases, TssK-RFPT assembles into spots without TssE-sfGFP (lower panel). Six representative fluorescence images are shown. Bars = 0.5 μm. **(B)** Aberrant phenotype of contractile sheath (TssB-GFP) in PA01Δ*tssE* strain. Fluorescence images from three representative time-lapse series of Δ*tssE* cells are shown. Note that the TssB-sheath is unusually elongated and curved and breaks off from the membrane. Bars = 0.5 μm. Schematic representation of bacteria with TssB (green) in PAO1Δ*tssE*.

TssE is the structural homolog of the T4 phage baseplate components gp25 (Leiman et al., 2009; Lossi et al., 2011). TssE oligomerises within the baseplate together with TssF and TssG, interacts with the sheath element TssB (Cherrak et al., 2018; Nazarov et al., 2018) and was proposed to act as an initiator of sheath polymerization (Kudryashev et al., 2015; Yap et al., 2010). To check contribution of TssE in TssK-mediated assembly of TssB-sheath in *P. aeruginosa*, we generated *P. aeruginosa* mutant Δ*tssE* expressing TssK-sfGFP and analyzed TssK-assemblies. TssK-sfGFP retained the capacity to assemble into foci in Δ*tssE* mutant and in the strain Δ*tssE/tssE* (Supplementary Figure S5). Similar to TssK-GFP, the TssE-sfGFP fusion protein also exhibited homogenous cytosolic localization in a majority of bacteria with a minor subpopulation of cells where TssE-sfGFP assembled into discernible perimembrane spots (Figure 5A). To confirm that TssK and TssE assemble into the same spot-like structure we generated bacteria co-expressing TssE-sfGFP with TssK-RFPT. Analysis of *P. aeruginosa* co-expressing TssE-sfGFP and TssK-RFPT (Figure 5A) revealed that all discernible TssE assemblies colocalized with TssK (upper panel) while not all TssK-RFPT assemblies were positive for TssE-sfGFP (lower panel). We generated also a Δ*tssE* strain expressing TssB-GFP. As already reported (Basler and Mekalanos, 2012; Vettiger and Basler, 2016), very low number of TssB-structures were seen in the mutant bacteria, and this was corrected by complementation (Supplementary Figure S5C). The time-lapse analysis of TssB-sheaths in Δ*tssE* revealed an aberrant phenotype, where the sheaths were unusually elongated and curved, not perpendicular to the membrane, and their dissociation from the inner membrane followed by disintegration into fragments was readily observed (Figure 5B), suggesting a weak or inefficient connection to the baseplate. This aberrant phenotype may explain the extremely low activity of the T6SS machinery in the absence of TssE observed in *Vibrio* (Basler and Mekalanos, 2012; Vettiger and Basler, 2016).

From these results, we conclude that (i) TssK component of the baseplate assembles independently of TssE, (ii) TssE is recruited to the baseplate complex at later stages as already suggested, and (iii) TssE is dispensable for TssB-sheath assembly *per se* but is required for stabilization of the connection between the sheath and the baseplate.

## Discussion

The access to assembly of multi-protein, membrane-bound architectures in live cells is extremely challenging in the context of spatial and temporal resolution. Here we focused on dynamic and regulatory aspects of assembly/disassembly of the T6SS baseplate components, TssK and TssE, and TssB by microscopy imaging of intact bacteria. We followed their capability to assemble into discernible higher order structure(s) depending on post-transcriptional regulatory module, their subcellular localization and spatial orientation and their dynamic characteristics (assembly/disassembly).

We found that TssK assembles into higher-ordered structure localizing to discrete foci at the cell periphery in the proximity of exogenous attacker and initiates further assembly of the contractile sheath, in agreement with its higher-ordered oligomerization capacity within the T6SS baseplate. In the complex baseplate-extended sheath forms TssK the connector ring between the membrane complex TssJLM and the rest of the baseplate. The overall structure has a six fold symmetry in the center, and is composed by two TssF interconnected by one TssG. TssG then contacts two TssK trimers trough extended loops to form a final structure containing 18 subunits of TssK (Cherrak et al., 2018; Nazarov et al., 2018; Park et al., 2018).

Based on those structural studies and observations by others, the C-terminal fusion construct is not sterically incompatible and should not impede TssK binding to its partners (TssL, TssM, TssG, and TssF). TssK-sfGFP used in our work is probably not competent for *E. coli* killing because of the GFP molecule hindering structural movements in TssK oligomer required to trigger final VgrG-toxin expulsion, although we could still observe the dueling.

The main finding in this study is the transient characteristic of TssK structures suggesting that they assemble *ad hoc* as structural entities and disassemble together with the contractile sheath TssB to recycle the components for *de novo* assembly of the apparatus in another site of the cell. Results of our kinetic analysis propose that TssK assembles upstream of TssE, the major transmembrane anchoring component TssM, and contractile sheath component TssB. It also requires presence of at least two essential components of the membrane-embedded sensory module, TagQ and TagR. This is in agreement with previous findings showing that TagQ-TagR-PppK-Fha-PppA pathway recognizes an external signal to activate the T6SS (Mougous et al., 2007; Basler et al., 2012; Ho et al., 2014). Our laboratory found that the sensory module in *P. aeruginosa* has additional components TagS and TagT that form an inner membrane ABC transporter with TagT having an ATPase activity (Casabona et al., 2013). Here we show that mutants in any of these two components are still capable to assemble TssK structures even if with lower incidence within the cell population. This opens a possibility that TagQ and TagR represent the core unit of the sensory complex able alone to trigger assembly of the TssK component of the baseplate whereas TagS and TagT probably through their ATPase activity may have a role in promoting higher-order oligomerization of proteins within the baseplate to adopt and stabilize the whole sheath/tube entity. TagT was indeed required for assembly of T6SS sheaths upon activation by polymyxin B treatment (Ho et al., 2013), or upon attack by a neighboring T6SS-positive bacteria (Basler et al., 2013), as assessed by the ClpV-GFP fusion. This observation could be a consequence of TagT effect on capacity of TssK to oligomerize in higher-ordered structures. We previously found that fusion construct TagQ-mCherry expressed in *P. aeruginosa* localizes to the outer membrane, but no discernible structures could be detected (Casabona et al., 2013). We thus hypothesize that multiple TagQRST modules may be scattered throughout the surface of bacteria and function as receptors sensing physical contract with kin or prey cells to trigger rapid assembly of the T6SS apparatus by recruitment of baseplate components at close proximity to assure effective attack toward a predatory species. Intriguingly, the *E. coli* TssK assemblies are static component of the baseplate (Brunet et al., 2015). This difference in TssK behavior may reflect the difference in regulatory features between *E. coli* and *P. aeruginosa* T6SSs, notably the absence of TagQRST-PpkA-PppA signaling in the *E. coli* system (Boyer et al., 2009).

What provides the link to the transmembrane anchoring complex of the T6SS once the baseplate begins to assemble? Previous studies using bacterial two-hybrid analysis identified direct interactions between TssK and TssM and between TssK and TssL, but only the TssK/TssL interaction was confirmed by co-immunoprecipitation (Zoued et al., 2013). In our study, we showed that membrane protein TssM is essential for lateral stabilization of TssK foci but not for the TssK assembly. It is possible that TssL alone assures TssK recruitment from the cytosolic pool into the perimembrane site. Biochemistry and bacterial two-hybrid screen revealed that TssK physically interacts with N-terminal cytoplasmic domain of TssL, residues 1–184 (Zoued et al., 2013). Moreover, study of T6-secretion in *A. tumefaciens* (Lin et al., 2014) proposed a model for the TssL-TssM complex where TssL is first phosphorylated by PpkA leading to ATP-binding dependent conformational switch in TssM followed by direct binding of FHA domain of Fha to the TssL. As thus the TssM-p-TssL-Fha complex powered by TssM-mediated ATP hydrolysis (Ma et al., 2012) may initiate recruitment of tube core component Hcp for assembly. Analysis of TssK behavior in specific mutants, either lacking domains of interactions between those components, mutants deficient in TssM ATPase activity, or bearing Fha unable to be phosphorylated, should bring more insights into molecular mechanisms governing the initial recruitments of TssK.

Our data suggest that although TssE assembles into a single perimembrane structure similar to TssK, it does so independently and downstream of assembly of TssK baseplate component. TssE homolog in bacteriophage T4, gp25, is essential to initiate assembly of the tube (Wagenknecht and Bloomfield, 1977). TssE assembles in the outermost structure of the baseplate hub around the membrane-puncturing device (Cherrak et al., 2018; Nazarov et al., 2018), as does gp25 (Kostyuchenko et al., 2003; Leiman et al., 2009). In contrast to TssK, we found that, in *P. aeruginosa*, TssE was not essential for TssB assembly into a sheath-like structure, although the sheath in a Δ*tssE* mutant exhibited an aberrant phenotype and break itself in smaller pieces, in agreement with almost undetectable activity of T6SS in competition assays found by others (Vettiger and Basler, 2016). In some Δ*tssE* bacteria where the TssB-sheath elongated parallel (or in a certain angle) to the membrane, the elongation of the apparatus was not spatially limited by cell width and TssB-sheath thus often assembled to an extreme length. This implies that although TssE and TssK co-assemble into a baseplate structure they adopt distinct functions where TssE incorporation into the baseplate may fortify the physical link between tube-sheath complex and the baseplate. Access to spatio-temporal behavior of two other baseplate proteins, TssG and TssF, should provide in the future global picture of the role of the baseplate in T6SS function.

**FIGURE 6:**
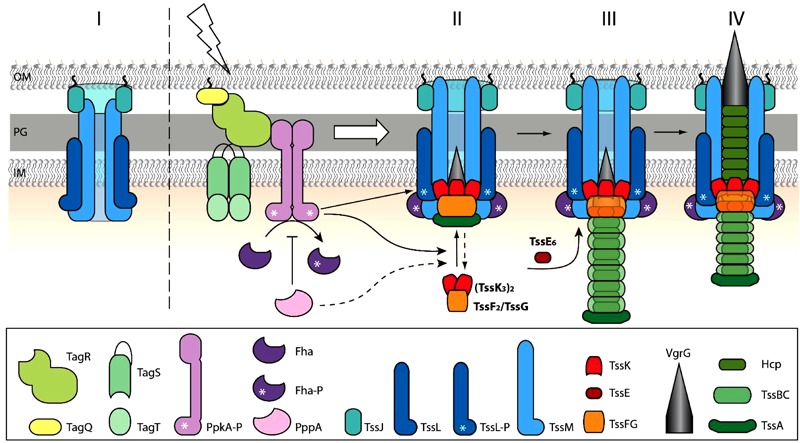
Schematic model for TssK/TssE baseplate assembly and function. **(I)** In a steady state, the baseplate components TssK and TssE are present in the cytosol. **(II)** Upon contact with target cell and activation of TagQRST (lightning) the Fha is phosphorylated by activated PpkA and binds TssL associated with the inner membrane. Upon activation of TagQ, TagR, and TssK oligomerizes, probably concurrently with TssF and TssG, around VrgG and the whole complex (VrgG(TssK_3_)_6_(TssF_2_/TssG)_6_) is recruited into the baseplate and anchored to the transmembrane complex TssJLM. The TssK oligomers disassemble upon action of PppA (shown in dashed lines). Stoichiometry (TssK_3_)_2_(TssF_2_/TssG) in accordance with cryo-EM structure (PDB 6N38) is shown. **(III)** Nucleation of the tube-sheath assembly begins by recruitment of Hcp-TssB/C subunits. TssE joins the TssF-TssG complex to form a more stable tube-sheath structure. **(IV)** The fully extended state is ready to “fire”. Outer membrane (OM), peptidoglycan (PG), inner membrane (IM).

In conclusion, our work shows the role of transmembrane post-translational regulatory cascade in activation and/or localization of T6SS baseplate assembly and the role of TssK and TssE in spatio-temporal organization and stabilization of T6SS contractile sheath in *P. aeruginosa*. Despite the fact that due to the low fluorescence intensities we could not examine the assembly of TssF and TssG, we present a model (Figure 6) where trimers of TssK associate with TssF/TssG complex transiently at the perimembrane site in contact with prey bacteria. The baseplate assembly occurs downstream of TagQRST activation and PpkA activity and functions as an essential spatio-temporal cue for subsequent recruitment of other components of the machinery including TssE, Hcp, and TssB/TssC leading to assembly of functional contractile apparatus with stable anchor within bacterial membrane. Our model also highlights the role of TssK in PppA-mediated recycling and repositioning of the machinery through *de novo* assembly at different perimembrane location defined by local activation of TagQRST module upon contact with the target cell.

## Materials and Methods

### Bacterial Strains and Genetic Constructions

All bacterial strains are listed in the Supplementary Table S1. Wild-type *Acinetobacter bauamnnii* and its Δ*tssM* mutant was kindly provided by Brent Weber (Alberta Glycomics Centre, University of Alberta, Canada) and described earlier (Weber et al., 2013). *P. aeruginosa* wild type and mutants Δ*tssM* (PA0077), Δ*tagQ* (PA0070), Δ*tagR* (PA0071), Δ*tagS* (PA0072), Δ*tagT* (PA0073), Δ*pppA* (PA0074), and Δ*ppkA* (PA0075) were kindly provided by J. Mougous Laboratory (University of Washington, United States) and described earlier (Silverman et al., 2011). Original plasmids and plasmid constructs used in this study are summarized in Supplementary Table S2. Fusion constructs *tssK*-sfGFP and *tssE*-sfGFP were generated as follows. First, the gene sequence encoding sfGFP [GenBank: HQ873313.1] was synthesized (Genscript, United States) to contain 5′ terminal *Spe*I and 3′ terminal *Sac*I restriction sites. This product was subcloned into pJN105 plasmid (Newman and Fuqua, 1999) via *Spe*I/*Sac*I restriction sites to generate pJN105-sfGFP. Second, genes encoding TssK (PA0079) and TssE (PA0087) without stop codon were amplified from *P. aeruginosa* PAO1 genomic DNA by PCR using primers listed in (Supplementary Table S3) and subcloned into pJN105-sfGFP via *EcoR*I/*Spe*I restriction sites. Fusion construct TssB-GFP was synthesized (ProteoGenix, France) to contain 5′ terminal *Eco*RI restriction site followed by 20nt 5′ TCGCGCGAGGGAGAAACAAG including ribosome-binding site, gene encoding TssB (PA0083), a linker sequence (5′ GCCGCCGCCGGCGGCGGC 3′), gene encoding GFP [GenBank: LN515608.1] and 3′ terminal *Sma*I restriction site. This synthetic product was subcloned into pJN105 via *Eco*RI/*Sma*I restriction sites to generate pJN105-*tssB*-*GFP*. Fusion construct *tssK*-*RFPT* was generated as follows. First, gene sequence encoding TagRFP-T (RFPT in this manuscript) [GenBank: EU582019.1] without ATG start codon was codon optimized for expression in *P. aeruginosa* and synthesized (Genescript, Unites States) to contain 5′ terminal *Spe*I restriction site followed by a linker sequence (5′ GCCGCCGCCGGCGGCGGC 3′), gene encoding TagRFP-T and 3′ terminal *Sac*I restriction site. This synthetic product was subcloned into pSW196 plasmid *via Spe*I/*Sac*I restriction sites to generate pSW196-RFPT. Second, gene encoding TssK was subcloned from pJN105-*tssK*-*sfGFP* into pSW196-*RFPT* via *EcoR*I/*Spe*I restriction sites to generate pSW196-*tssK*-*RFPT*. Plasmids encoding fusion constructs were introduced into *P. aeruginosa* strains by heat-shock transformation for pJN105-derived plasmids or by triparental mating for pSW196 integrative plasmids. To generate an in-frame deletion of *tssE* (PA0087) in *P. aeruginosa*, sequences corresponding to upstream and downstream flanking regions of the genes was synthesized (Genscript, United States) to contain *Sac*I and *Xba*I restriction sites on 5′ and 3′ terminus, respectively. This synthetic product was subcloned into pEXG2 plasmid (Rietsch et al., 2005) for subsequent *sacB*-mediated allelic exchange as described earlier (Metcalf et al., 1996). Deletion was confirmed by PCR using primers listed in Supplementary Table S3. The similar level of expression of fusions in different strains was confirmed by immunoblotting (Supplementary Figure S3). For complementation experiments, gene of interest was cloned into a pminiCTX1-derivative plasmid pSW196 containing arabinose-inducible promotor pBAD. Then, pSW196 plasmid containing *tssE* or *tssK* gene was introduced in *P*. *aeruginosa* by triparental mating (Hmelo et al., 2015).

### Competition Assays

The competition assays were performed as previously described (Hachani et al., 2013). *P. aeruginosa* and *E. coli* (pBlueScript) were grown overnight in 3 ml LB medium supplemented with appropriate antibiotics. Diluted overnight cultures were inoculated in the same medium containing 0.025% arabinose (to induce the expression of specific fusion) and the culture was grown until OD_600_ = 1. Then 1 ml of each culture were spin down and pellet was resuspended in 100 μl LB with arabinose 0.025%. Indicated *P. aeruginosa* strains (predator) were mixed with *E. coli* (prey) in ratio 1:2 (predator:prey). Competition reactions (20 μl), realized in triplicate, were spotted onto LB agar plates containing 50 μg/ml ampicillin and incubated 5 h at 37°C. The totality of bacteria was recover in LB and dilutions were plated in triplicates onto LB plates containing Xgal (40 μg/ml) and IPTG (100 μM) to visualize *E. coli* (blue colonies).

### Western Blotting

The expression levels of protein fusions in different mutants were assessed by immunoblotting. For the Western blot, total bacterial samples (OD_600_ = 1) were separated on Criterion 4–20% TGX precasted gels, BioRad and transferred onto a PVDF membrane (GE.Healthcare) by electrotransfert in 20% Laemmli buffer. After blocking step in 5% milk, polyclonal anti-GFP antibodies (diluted 1/5000 in PBS buffer with 0.1% Tween20) were incubated one hour at room temperature, followed with a second antibodies incubation (anti-rabbit HRP, dilution 1/20 000, Sigma). Detection was performed using Luminata Classico HRP-substrate (Millipore) using BioRad ChemiDoc apparatus.

### Time Lapse Video Microscopy

Bacteria were grown over night in LB with appropriate selection antibiotics, then sub-cultured 1:100 in LB with arabinose (0.025–0.25%) for induction of fusion construct expression and grown for additional 3 h to reach OD_600_ = 2. To immobilize bacteria for acquisition of still images or a time-lapse series, a drop of bacterial culture was applied on top of agarose pads [1.5% Low melting agarose in Hanks’ balanced salt solution (Gibco)] and transferred into the glass-bottom imaging dishes (WillCo Wells BV) so that bacteria were grown in interphase between glass bottom and semi-solid medium of agarose pad for another 1 h prior imaging. For competing conditions, culture of *P. aeruginosa* was mixed 1:1 with competing bacteria (*A. baumannii*) prior applying onto agarose pads. Still images or time-lapse image acquisitions was performed within 1–3 h post mixing competing bacteria – a time frame when we observed the peak activity of TssK/TssB assemblies. Fluorescence and bright field images were acquired at a rate of 6 frames per min (10 s between frames) for 5–15 min using OLYMPUS inverted microscope IX71 equipped with UPlanFLN 100× NA1.3 objective and 16-bit Hamamatsu ORCA-ER CCD camera, operated by Xcellence RT2.0 acquisition software (OLYMPUS). Digital image processing and analysis including measurements of fluorescent intensity and kinetic profiles was made using ImageJ 1.47v (Wayne Rusband, NIH, United States). Where necessary, the *x*–*y* drift in time-lapse series was corrected by StackReg plugin using Rigid body transformation algorithm. Statistic evaluation was done in MS Excel 2010.

### Foci Quantification

Because rarely more than one discernible fluorescent structure of the fusion construct of TssK or TssB was detected per cell at a time, still images of live bacteria were used to quantify the incidence of assembly as a fraction (%) of cells that exhibit discernible fluorescent structure relative to the total amount of bacteria in the view field. For every sample and condition analyzed, a set of images was acquired from random fields of view using fixed exposure settings to allow quantitative comparison between samples. Because the expression levels of the fusion construct was usually heterogeneous within the same population of bacteria, automated particle counting on binary images using thresholding, filtering and segmentation algorithm was not reliable and thus manual counting from images was used instead after 16-bit to 8-bit conversion and background subtraction. Average and SD values were calculated for each group from 4 to 6 ROI each containing between 400 and 1000 of cells.

### Kinetic Measurements of Fluorescence Intensity

Kinetic changes of fluorescence intensity were measured on a stack of images from a time-lapse. First, a set of ROI of fixed size (500×500 nm) covering area where discernible TssK/TssB-structures appear during the time-lapse was selected and fluorescence integrated density within these ROI was measured using ROI manager and multiple measurements plugin (ImageJ). Illustrative results for a typical profile were plotted as a fold-increase/decrease in fluorescence intensity relative to ROI intensity in frame 1 (T0) of the time-lapse (set as nominal value 1). Average duration of assembly was assessed as a time between the onset of fluorescence increase over the background level (rel. value = 1.0) and the peak fluorescence values. On contrary, average duration of disassembly was assessed as a time between fluorescence peak and its decrease to the background levels. Average values and SD were calculated from 20 to 40 ROI for each group. For an assessment of spatio-temporal colocalization between TssK-RFPT and TssB-GFP the fluorescence intensity for both channels (RFPT and GFP) was first measured at multiple ROI of several independent time lapse series. The resulting kinetic plot profiles were then aligned along *y*-axis so that maximum peak values for GFP channel (fully extended TssB-sheath) fit to *T* = 0 s and fluorescence intensity values of RFPT corresponding to each time point of GFP values were plotted accordingly. Average values and SD were calculated from 20 kinetic profiles for each channel.

### Fluorescence Intensity Measurements of Protein Assemblies

For measurement of fluorescence intensity profile of TssK and TssB structures (Figure 1A) images were acquired with fixed exposure settings and fluorescence intensity plot profile was measured on line cross-section through the structures. Maximum fluorescence intensity (Max gray values) of TssK spots in *tagS* and *tagT* mutants (Figure 1C) was measured by ImageJ in fixed-size square ROI drawn around the fluorescent spots. Average and SD values were calculated from 20 plot profile measurements for each group. For an assessment of spatial co-localization between TssK-RFPT and TssB-GFP in elongated TssB-sheaths, fluorescence intensity plot profile was measured along the long axis of the structure with proximal end of the ROI crossing perimembrane TssK structure.

## Data Availability

The raw data supporting the conclusions of this manuscript will be made available by the authors, without undue reservation, to any qualified researcher.

## Author Contributions

DL, MR-G, VJ, and VC performed the experiments and prepared the figures. DL and IA designed the study, analyzed the data, and wrote the manuscript.

## Conflict of Interest Statement

The authors declare that the research was conducted in the absence of any commercial or financial relationships that could be construed as a potential conflict of interest.
