# Supplementary material for: Baseplate Component TssK and Spatio-Temporal Assembly of T6SS in Pseudomonas aeruginosa
Source: Front Microbiol. 2019 Jul 18;10:1615. doi: 10.3389/fmicb.2019.01615 (PMC6657622; doi:10.3389/fmicb.2019.01615)
Supplement: Supplementary file 1 [file Data_Sheet_1.pdf]

Supplementary material

Liebl et al.

Supplementary Tables

Table S1. List of bacterial strains and mutants

Bacterial strain	Description	Source or reference
P. aeruginosa PAO1	PAO1-UW	J. Mougous Lab
	Δ*pppA* (PA0075)	(51)
	Δ*ppkA* (PA0074)	(51)
	Δ*tagQ* (PA0070)	(51)
	Δ*tagR* (PA0071)	(51)
	Δ*tagS* (PA0072)	(51)
	Δ*tagT* (PA0073)	(51)
	Δ*tssM* (PA0077)	(51)
	Δ*tssE* (PA0087)	this work
	Δ*tssK* (PA0079)	this work
A. baumannii 17978	WT	(40)
	Δ*tssM*	(40)
E. coli	DH5α pRK2013	Laboratory collection
E.coli	DH5α pBluescript	Laboratory collection

Table S2. List of plasmids

Name	Description and relevant features	Source or reference
pJN105	*Replicative plasmid*<br>*(araC*-p*BAD* cloned in pBBR1MCS-5; Gm^R)	(52)
pSW196	mini-CTX1 based plasmid<br>*(araC*-p*BAD* from pBAD30; Tc^R)	(56)
pRK2013	helper plasmid for conjugation<br>(oriColE1 RK2-Mob+ RK2-Tra+; Km^R)	Addgene
pEXG2	Cloning vector for allelic exchange, *sacB*, Gm^R	(53)
pCR-Blunt-II-Topo	Plasmid for fusion gene subcloning and sequencing	Invitrogen
pJN105:TssK-sfGFP	C-Term. PA0079 translational fusion with sfGFP	This study
pJN105:TssB-GFP	C-Term. PA0083 translational fusion with GFP	This study
pJN105:TssE-sfGFP	C-Term. PA0087 translational fusion with sfGFP	This study
pSW196:TssK-RFPT	C-Term. PA0079 translational fusion with RFPT	This study

Table S3. List of primers

Primers	Sequence	Description
TssK-F	5'CCGAATTCACCTTCGGAGTCCCTATGTCC'3	PCR PA0079/TssK
TssK-R	5'CCACTAGTTCCTCGAATGGCCCAGAAGGC'3	
TssK.TGA-R	5' CGACTAGTTCATCCTCGAATGGCCC 3'	
TssE-F	5'CCGAATTCGCCGACGGACGCCTGACATG'3	PCR PA0087/TssE
TssE-R	5'CCACTAGTTGTACGCCTCCGCTCGCCCT'3	
TssE.TGA-R	5' CGACTAGTTCATGTACGCCTCCGCTC 3'	

Supplementary Figures

FIGURE S1

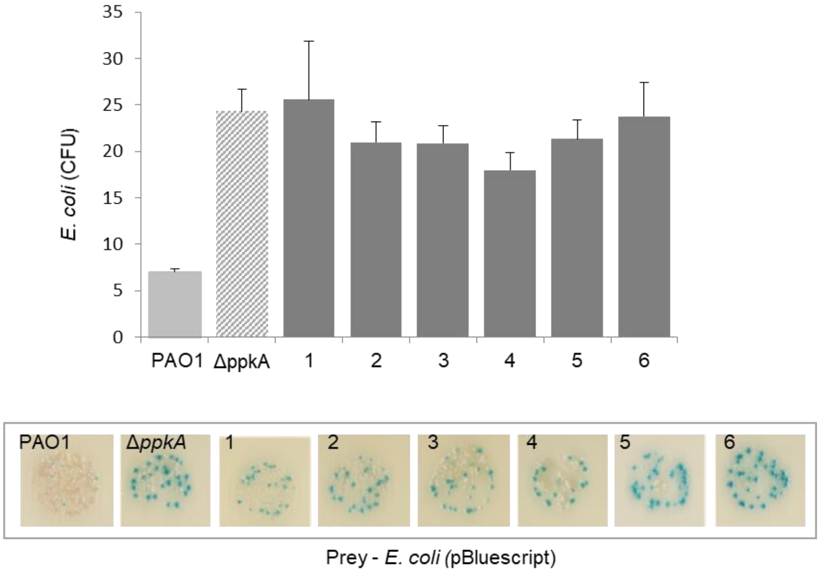

Figure S1. Functionality of T6SS analysed by competition assays between PAO1 strains expressing different fusion constructs and *E. coli* DH5α containing pBlueScript. PAO1 (WT) expressing RFPT (from pSW196) and PAO1Δ*ppkA* were used as positive and negative control, respectively. PAO1 expressing (1) *tssK-sfGFP*, (2) *tssE-sfGFP*, (3) *tssB-GFP*, (4) *tssB-GFP* and *tssK-RFPT*, (5) *tssE-sfGFP* and *tssB-RFPT* and (6) *tssE-sfGFP* and *tssK-RFPT* were tested against *E. coli* as a prey. All fusions are described in Material & Methods. Expression of fusions were induces with 0.025% arabinose. Competition assays show that all fusions inhibit T6SS-mediated killing compared to the PAO1 WT. Upper panel: counts of *E. coli* colony forming units (CFU) in three independent competing reaction. Lower panel: representative images of 10^{-5} dilution spotted on LB, X-gal/IPTG plates.

FIGURE S2

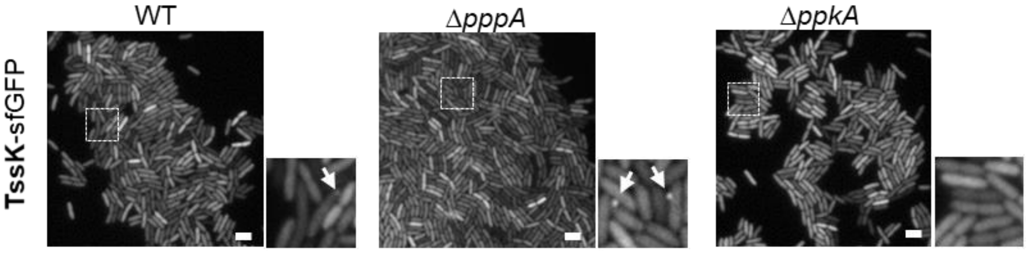

Figure S2. Fluorescence microscopy images of *P. aeruginosa* wild-type, Δ*pppA* and Δ*ppkA* mutants expressing TssK-sfGFP. Note that no discernible TssK-sfGFP assemblies were found in Δ*ppkA* mutant cells. Representative fluorescence images are shown. TssK-spots are highlighted by arrows. Bars = 0.5 µm

FIGURE S3

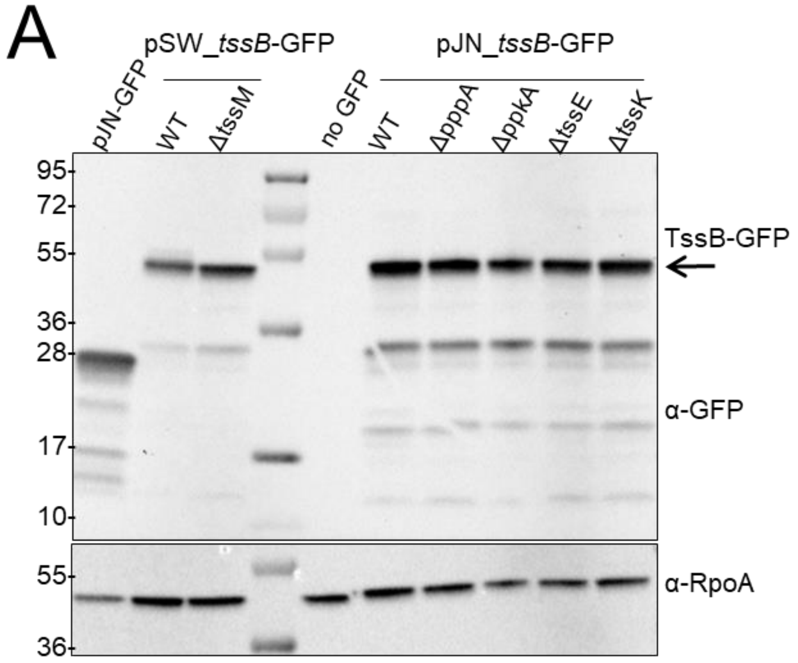

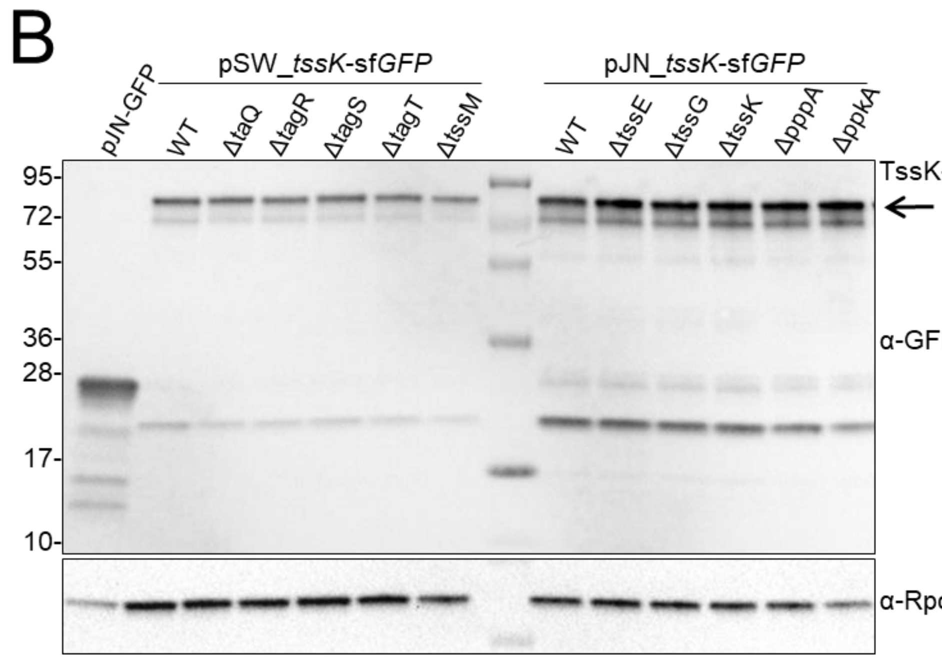

Figure S3. Expression levels of different protein fusions with sfGFP in wild-type strain and different mutants. Strains expressing *tssB-sfGFP* (A) or *tssK-sfGFP* (B) from either integrative

pSW196 or replicative pJN105 plasmid were analyzed by immunoblotting using GFP polyclonal antibodies. RpoA was used as loading control.

FIGURE S4

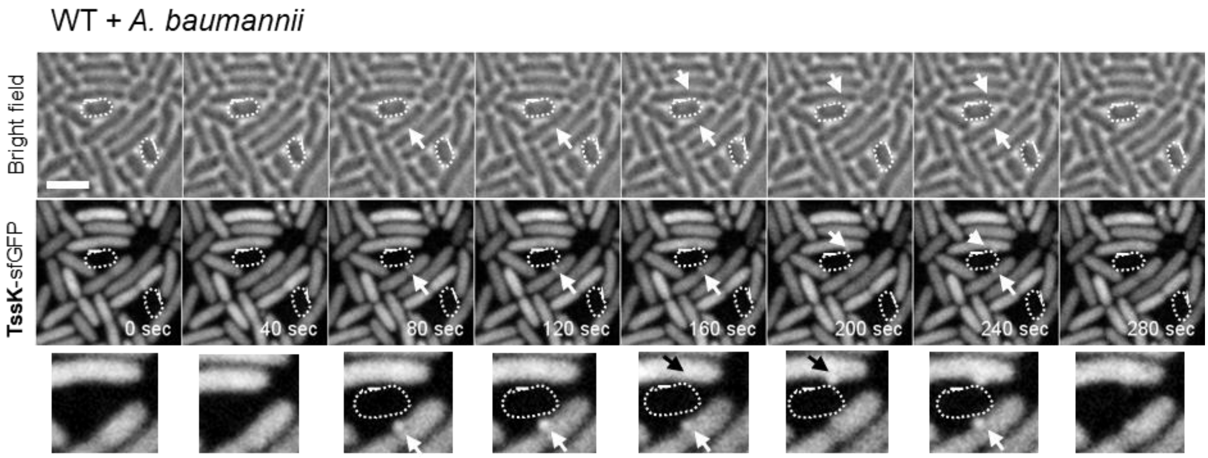

Figure S4. Time-lapse series of *P. aeruginosa* expressing TssK-sfGFP in mixed culture with *A. baumannii* (border by dashed lines). Note that perimembrane TssK spots are oriented specifically towards the contact with competing bacteria (arrows). Selected bright field (upper panel) and fluorescence (lower panel) images from a time-lapse are shown. Bars = 0.5 µm

FIGURE S5

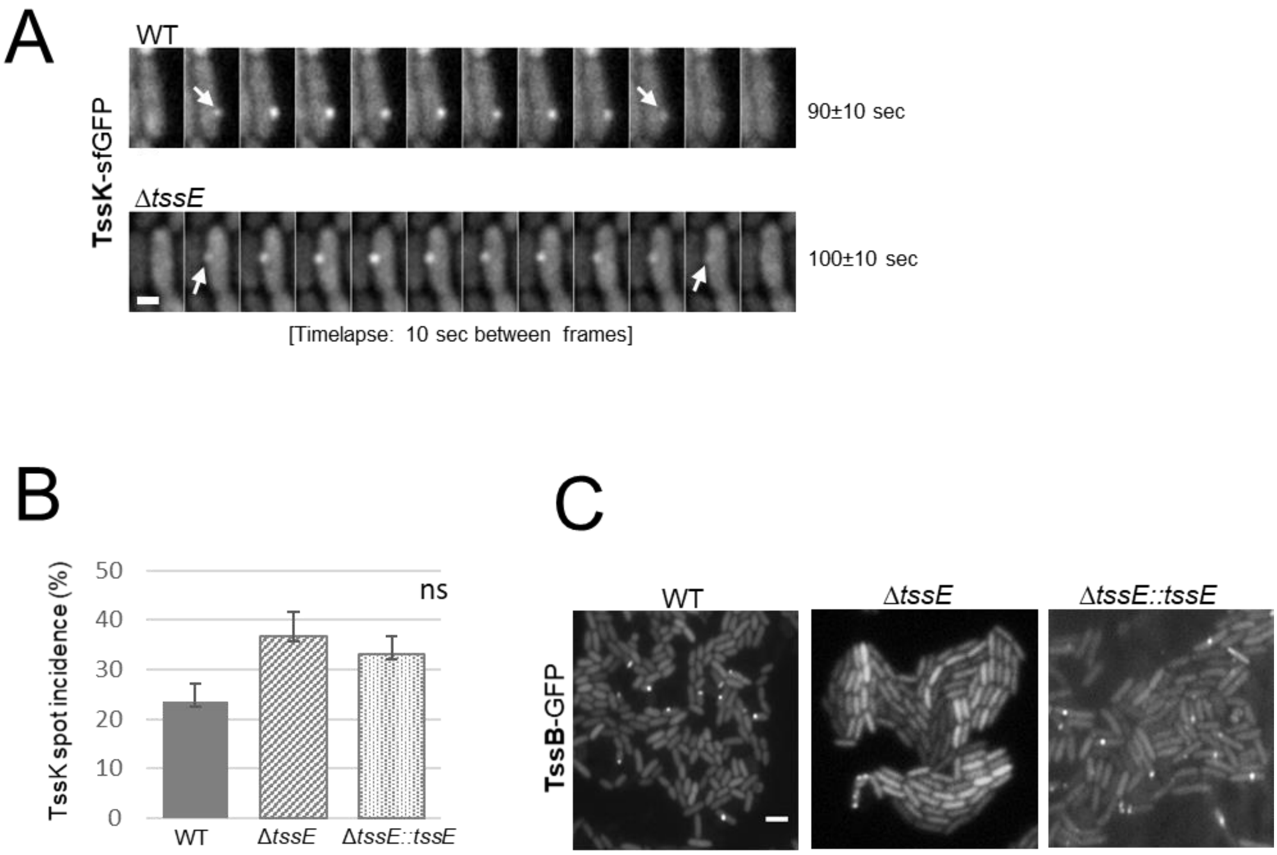

Figure S5. (A) Dynamics of TssK-baseplate assembly-disassembly is not significantly affected in Δ*tssE* mutant in comparison to the wild-type *P. aeruginosa* PAO1. Time-lapse series demonstrate transient, perimembrane assembly and disassembly of TssK-baseplate structure (indicated by arrows) within a period of 90-100 sec. Bars = 0.5 µm. (B) TssK spot incidence in the wild-type PAO1 strain, in Δ*tssE* and in complemented strain Δ*tssE/tssE* expressing the same construct was not significantly different. (C) Assembly of TssB-GFP structures in wild type (WT), Δ*tssE* and Δ*tssE*::*tssE*. Note readily detectable TssB structures in WT and Δ*tssE*::*tssE.*

Supplementary Material and Methods

Competition assays

The competition assays were performed as previously described (Hachani *et al*., 2013). *P. aeruginosa* and *E. coli* (pBluescript) were grown overnight in 3 ml LB medium supplemented with appropriate antibiotics. Diluted overnight cultures were inoculated in the same medium containing 0.025% arabinose (to induce the expression of specific fusion) and the culture was grown until OD_{600}=1. Then 1 ml of each culture were spin down and pellet was resuspended in 100 µl LB with arabinose 0.025%. Indicated *P. aeruginosa* strains (predator) were mixed with *E. coli* (prey) in ratio 1:2 (predator:prey). Competition reactions (20 µl), realized in triplicate, were spotted onto LB agar plates containing 50 µg/ml ampicillin and incubated 5 hours at 37°C. The totality of bacteria was recover in LB and dilutions were plated in triplicates onto LB plates containing Xgal (40 µg/ml) and IPTG (100 µM) to visualize *E. coli* (blue colonies).

Western blotting

The expression levels of protein fusions in different mutants were assessed by immunoblotting. For the Western blot, total bacterial samples (OD_{600}=1) were separated on Criterion 4-20% TGX precasted gels, BioRad and transferred onto a PVDF membrane (GE.Healthcare) by electrotransfert in 20 % Laemmli buffer. After blocking step in 5 % milk, polyclonal anti-GFP antibodies (diluted 1/5000^{e} in PBS buffer with 0.1 % Tween20) were incubated one hour at room temperature, followed with a second antibodies incubation (anti-rabbit HRP, dilution 1/20 000, Sigma). Detection was performed using Luminata Classico HRP-substrate (Millipore) using BioRad ChemiDoc apparatus.

Supplementary reference

1. Hachani A, Lossi NS, & Filloux A (2013) A visual assay to monitor T6SS-mediated bacterial competition. *Journal of visualized experiments : JoVE* 10.3791/50103(73):e50103.
